# Identification of Perception Differences in Personality Factors and Autonomy by Sporting Age Category in Competitive Bodybuilders

**DOI:** 10.3390/ijerph20010167

**Published:** 2022-12-22

**Authors:** Elena Mihăiță, Dana Badau, Marius Stoica, Georgeta Mitrache, Monica Iulia Stănescu, Ioszef Laszlo Hidi, Adela Badau, Cosmin Damian, Mirela Damian

**Affiliations:** 1 Doctoral School, National University of Physical Education and Sport, 060057 Bucharest, Romania; 2 Petru Maior Faculty of Sciences and Letters, George Emil Palade University of Medicine, Pharmacy, Sciences and Technology, 540142 Targu Mures, Romania; 3 Interdisciplinary Doctoral School, Transilvania University, 500068 Brasov, Romania; 4 Faculty of Physical Education and Sport, Ovidius University, 900527 Constanta, Romania

**Keywords:** personality, autonomy, bodybuilding, competitive bodybuilders, sporting age categories

## Abstract

Knowing the personality and autonomy factors of bodybuilders is a necessity in order to improve sports training, which would lead to the development of mental skills specific to competitive bodybuilding. The purpose of the study was to identify perception differences in personality and autonomy factors for three age categories of competitive bodybuilders in order to optimize the sports training process. The secondary purpose of the study was to identify perception differences in the characteristics of personality and autonomy in relation to the increase in the sporting age category of the competitive bodybuilders. The research sample included 30 competitive bodybuilders aged between 18 and 53 years, of which 21 were male and 9 were female, who were divided into three groups according to the sporting age category: G1—junior (18–23 years old), 10 athletes (33.3%); G2—senior (24–35 years old for female and 24–40 years old for male), 12 athletes (40%); G3—masters (over 35 years old for female and 40 years old for male), 8 athletes (26.7%). In the precompetitive stage, three standardized questionnaires were applied to the study participants, namely, two personality questionnaires (CP5F and ZKPQ) and a personal autonomy questionnaire (PAQ), which provided us with useful information for understanding the psychological profile of bodybuilding athletes. The results of the study were statistically significant, with Cronbach’s Alpha coefficient indicating high internal consistency of the three questionnaires for all three sporting age groups, with higher values than the selected reference threshold of 0.700. According to the results of our study, the following personality and autonomy factors recorded higher and higher scores in direct relation with the increase in sporting age: extraversion, agreeableness, conscientiousness, emotional stability, autonomy, impulsive sensation seeking, value autonomy, behavioral autonomy, and cognitive autonomy. The personality and autonomy factors of which the scores did not increase directly proportionally to the sporting age category were sociability, activity, aggression–hostility, neuroticism–anxiety, and emotional autonomy. Competitive bodybuilders perceive the factors of personality and autonomy differently in relation to particularities of age and sports experience, and knowledge of these changes can contribute to the improvement of the sports training process

## 1. Introduction

Competitive bodybuilding aims to improve the esthetic and physical condition of the body through the symmetrical and harmonious development of muscle mass. The training process of bodybuilders includes specific exercises that use weights and specialized equipment. Athletic performance is influenced by age characteristics, anthropometric features, and the level of sports training [1,2]. Competitive sport requires complex preparation based on the intercorrelation of specific training factors, more precisely, physical, technical, tactical, psychological, and theoretical factors [3,4,5,6]. The International Fitness and Bodybuilding Federation (IFBB) has a history of over 70 years since its foundation, including over 200 national federations. In the last decade, competitive and recreational bodybuilding has registered an increasing dynamic of popularity, with the number of licensed athletes increasing annually [7]. The Romanian Federation of Bodybuilding and Fitness was founded in 1990, being an active member of the European Federation of Bodybuilding and Fitness (EBFF), the International Federation of Bodybuilding and Fitness (IFBB), and the Balkan Federation of Bodybuilding and Fitness [8]. In Romania, competitive bodybuilding is a legislatively recognized sport, organized on two types of structures: competitive bodybuilding (with a nationally and internationally recognized sporting activity) and recreational bodybuilding. A constant preoccupation of sports psychologists and experts in the sports field consists of identifying the methods of influence and the relationship between sports training factors, including physical, technical, and psychological preparation in order to optimize competitive sports performances. Recent studies have shown that psychological training plays a decisive role in developing the psycho-behavioral activities of an athlete, which generate effective behavior during both training and competitions [9,10,11]. Sports performance is the outcome of the intercorrelation of physical, technical, and psychological training [12,13,14]. Sports psychology, such as the important component of general psychology, focuses on the development of the personality and behavior of athletes. Sports psychology aims to identify the way in which the practice of sports activity contributes to the personal development of athletes, behavioral modeling, and other psychological dimensions including cognitive, emotional, motivational, and sensory-motor [15,16,17]. Mental training and psychological counseling are today common processes that are directed and monitored by sports psychologists in order to optimize the personality and behavior of athletes for the efficiency of the sports training process [18,19,20].

The identification and impact of personality factors in elite bodybuilders is a topic of great interest for sports psychologists, coaches, and athletes to understand both the complex process of achieving success in sport and the sporting life motivations [21,22,23,24]. Studies have shown that psychological training can make a difference in personal development and in the formation of a competitive psychologist in relation to the requirements of the sport practiced and the level of the sports category [25,26,27,28]. Psychological training determines the formation of an athlete’s personality and capacity for self-management, autonomy, and self-regulation [29,30]. It differs according to the sports training stage as follows: basic psychological training, psychological training for a specific sport, and psychological training for participation in sports competitions [31,32]. The components of psychological sports training are complex, interrelated, and dependent on athletes’ individual and sporting characteristics, and they include psychomotor training, cognitive training, volitional training, and emotional training [33,34,35].

Studies have highlighted that by carrying out a long formative process, such as sports training, it is possible to optimize characteristic psycho-neurological manifestations, behavior, temperament, and even personality as elements of the human genotype [36,37,38]. The development of personality traits is a major objective of the sports training process and establishing an athlete’s psychological profile includes intrinsic and extrinsic motivation, psycho-behavioral and psychomotor attitudes, temperamental characteristics, character traits, autonomy, self-awareness, etc. [39,40]. An athlete’s personality is built during the educational and sports training process, being influenced by biological, psychological, social, and cultural factors [40,41,42,43,44]. In competitive psychological sports practice, personality is analyzed and approached as a whole. The concerns of experts in psychology and sports psychology have been focused on the classification/systematization of the main five variables in order to establish a personality model, which resulted in the Big Five Model [45,46]. The Big Five Model was later expanded and diversified to also include other relevant factors that could influence and complete the human personality and therefore that of athletes [33,47]. Based on recent studies, personality profiles have been developed according to the type of sport practiced and the methodological criteria of the Big Five Personality Model, for both its classic version and alternative version [48,49,50,51].

The specifics and characteristics of the practiced sport condition the staged and complex selection process regarding anthropometric parameters, physical and technical abilities, certain characteristics of personality, and autonomy in order to select subjects with the best sporting potential [52,53,54,55,56]. Competitive sport requires the optimal development of an athlete’s personality and autonomy, the dynamics of which depend on age characteristics, level of training, and sports experience. Autonomy is reflected in a person’s ability to control their own activity and is formed as a result of experimentation and adaptation to various contexts [57]. In sport, autonomy is expressed by the athletes’ abilities to both set and make their own decisions about performance goals and to self-manage their performance through self-analysis and self-regulation of the sporting behavior in its entirety [24,58]. The development of autonomy in sport can be achieved by expanding communication and active and positive listening between staff and athlete, by presenting the perspectives of sports training, by getting actively involved in making decisions about the management of one’s own performance and sporting life, by prioritizing motivations, etc. [59,60].

Much research has focused on psychological training in competitive sport [61,62], but studies on how the athletes’ perceptions of the dynamics of personality and implicit autonomy undergo changes in relation to the sporting age category and the athletic experience are relatively few and do not address competitive bodybuilders [63,64,65]. Identifying the perception differences regarding the characteristics of personality and autonomy according to the sports age category, we consider that it will facilitate the expansion of knowledge, highlighting the importance of sports psychology in improving the training process of competitive bodybuilders.

The purpose of the study was to identify perception differences in personality and autonomy factors for three age categories of competitive bodybuilders in order to optimize the sports training process. The secondary purpose of the study was to identify perception differences in the characteristics of personality and autonomy in relation to the increase in sporting age category of competitive bodybuilders. The main hypothesis of the study started from the assumption that the characteristics of personality and autonomy are perceived differently by competitive bodybuilders in relation to the sports category of age and sports experience with an impact on the improvement of the training process. The knowledge by sports psychologists and coaches of the perception of personality and autonomy factors by competitive bodybuilders will facilitate the scientific management of training by understanding the psychological and behavioral aspects of athletes.

## 2. Materials and Methods

### 2.1. Study Design

The study was conducted from 18 August to 8 September 2021, in the precompetitive period of the National Bodybuilding and Fitness Championship organized between 9 and 11 September in Sibiu. The questionnaires were selected at the recommendation of sports psychologists, who argued that developing a complex psychological profile of the competitive bodybuilders should also incorporate the personality and autonomy dimensions. Studies have confirmed the multidimensional approach to the personality of elite athletes, recommending the parallel analysis of the three questionnaires selected for this research [66,67,68,69,70,71]. Sports psychologists consider that the personality of athletes must be approached and evaluated under the aspect of as many factors as possible in order to capture the complexity of athletes; in this session, sports psychologists recommend the simultaneous application of the three questionnaires selected for this study [66,67,68,69,70,71,72,73,74]. 

The questionnaires were applied by sports psychologists and the authors of the study. Bodybuilders were contacted directly or by phone in order to agree to participate in the study. Through a video meeting on Google Meet, the bodybuilders were informed about the content, structure of the questionnaire, and the method of completing and evaluating the items. The questionnaires were edited online in Google Form and were competed online by the athletes in the presence of the authors using laptops/tablets. After completing the questionnaires, the results were centralized for statistical processing.

### 2.2. Participants

The ascertaining study involved 30 competitive bodybuilders, of which 21 were male (70%) and 9 were female (30%). The 30 participants represented 95% of the established competitive bodybuilders in Romanian. All athletes included in the study are active, have relevant sports records, are qualified, and participate in national and international bodybuilding competitions. The sample was divided into three sporting age categories in compliance with the provisions of international bodybuilding regulations as follows:

- G1—juniors aged 18–23, consisting of 10 athletes (33.3%): 4 female and 6 male; average years of competitive experience: female 3 years, male 5 years;
- G2—seniors aged 24–35 for female and 24–40 for male, consisting of 12 athletes (40%); 4 female and 8 male; average years of competitive experience: female 7 years, male 11 years;
- G3—masters aged over 35 for female and 40 for male, consisting of 8 athletes (26.7%); 3 female and 5 male; average of years competitive experience: female 16 years, male 21 years.

Inclusion criteria: active athletes qualified for the National Bodybuilding and Fitness Championship organized between 9 and 11 September 2021 in Sibiu, participants in bodybuilding competitions in the last 6 months, optimal health status, sports records as at least national champions, full completion of the questionnaires. Exclusion criteria: training discontinuity in the last 6 months due to illness or injury, non-qualification or non-participation in competitions in the last 6 months, returning incomplete questionnaires. The optimal state of health aims at the acceptance of the sports doctor for participation in trainings and competitions. The athletes were asked about the periodic medical visa and about injuries, illnesses, and treatments in the last 6 months. In the study were included only athletes with up-to-date medical visas and with the status of clinically healthy, as approved by the sports doctor. This aspect was relevant for the study, because a poor state of health determines the interruption of the preparation process and participation in competitions, and this situation can influence the mental state of bodybuilders and implicitly their perception of the factors analyzed in this study. Participation in the test was voluntary and based on the informed consent of each athlete. The study complies with the principles of the Declaration of Helsinki and was approved by the Ethics Board of the National University of Physical Education and Sport in Bucharest, Romania, no. 2089/01.10.2020.

### 2.3. Assessment

Two personality questionnaires were used in the study, which complement each other and integrate different and relevant characteristics for the investigated athletes, but also a personal autonomy questionnaire that provided important information for understanding the psychological profile of bodybuilding athletes. We mention that the questionnaires applied in this study were validated for the Romanian language [68,69,70,71,72,73,74]. 

Description of the study questionnaires:

- Five-Factor Personality Questionnaire (CP5F)—assesses extraversion, agreeableness, conscientiousness, emotional stability, and autonomy, being validated for the Romanian language [68,69,70,71]. CP5F is a Romanian adaptation of the standardized Five-Factor Personality Inventory (FFPI), which aims to assess the five factors of the Big Five Model [68,69,70,71,72]. The questionnaire was structured into the five subscales (extraversion 23 items, agreeableness 24 items, conscientiousness 25 items, emotional stability 21 items, autonomy 22 items), which were rated on the Likert scale as follows: 1 point—suits me; 2 points—it fits me a little; 3 points—fits me about halfway; 4 points—suits me a lot; 5 points—suits me very well [68,69,70,71].
- Zuckerman–Kuhlman Personality Questionnaire (ZKPQ)—investigates the five factors of the Alternative Five-Factor Model (AFFM). The questionnaire was structured into the five subscales (impulsive sensation seeking 19 items, sociability 17 items, neuroticism–anxiety 19 items, aggression–hostility 17 items, activity 17 items), which were rated on the Likert scale as follows: 1 point—suits me very little, 2 points—suits me less, 3 points—suits me about half, 4 points—suits me much, 5 points—suits me very much [58,59,60,61,62,63,64,65,66,67,68,69,70,71,72,73].
- Personal Autonomy Questionnaire (PAQ)—assesses a person’s ability to control their own life and the feeling that there is a possibility to exercise this control [73,74]. The questionnaire consisted of 36 items and was structured into four subscales (cognitive autonomy 9 items, behavioral autonomy 11 items, emotional autonomy 8 items, value autonomy 8 items), which were rated on the Likert scale as follows: 1 point—very little, 2 points—a little, 3 points—neither too much nor too little, 4 points—much, 5 points—very much [73,74].

T-scores were used to interpret the results obtained by the study participants (taking into account their gender and age) on the personality test scales. Standardized gender and age-group samples are valid for individuals included in the nonclinical population and allow the conversion of raw scale scores into T-scores. A T-score is low if it is less than 40 and high if it is above 60. Average T-scores are in the range of 40 to 60 [69]. The questionnaire is used in various fields, including sports, education, and health, and for personality diagnosis [73].

Individual results, more specifically the responses to all items of the three personality questionnaires administered to each athlete, were entered into the CAS++ (Cognitrom Assessment System) application [69]; the system automatically generated an assessment report containing full information about the athletes, namely, (anonymized) identification data, qualitative information entered, results obtained on the administered psychological scales (raw scores, rated scores, graphical representations, and generic interpretations. CAS++ is a computerized platform for comprehensive psychometric and non-psychometric assessment, which uses accurate psychometric tools, meaning validated psychological tests. From the assessment reports generated for each athlete, the following data were extracted to be statistically processed: age, individual scores per item, rated scores per subscale, total scores per questionnaire.

### 2.4. Statistical Analysis 

The results of the study were statistically processed using SPSS 2022 software. To highlight the correlations between the results, the following statistical parameters were used: mean (X), standard deviation (SD), Student test (t), and the two-level (lower/upper) confidence interval (95% CI). Principal component analysis (PCA) was calculated for Bartlett’s Test of Sphericity (Chi-square) and the Kaiser–Meyer–Olkin (KMO) Test. For the reliability or internal consistency of the questionnaires, Cronbach’s Alpha index was calculated, and the lower bound was 0.70 for all measures. The mean difference between the three independent age groups was analyzed using analysis of variance (ANOVA). The value of the statistical reference significance was *p* < 0.05.

## 3. Results

The relevant results obtained by the three sporting age categories of competitive bodybuilders were structured and presented according to the three questionnaires and the study objectives.

According to Table 1, Cronbach’s Alpha scores reflected high internal consistency of the Five-Factor Personality Questionnaire (CP5F) for all three sporting age groups, with higher values than the selected reference threshold of 0.700. The same table reveals that the statistical values of the Kaiser–Meyer–Olkin parameter were above Kaiser’s criterion of >0.5 for all three sporting age groups. Analyzing the results of this questionnaire, it was found that Bartlett’s Test of Sphericity values showed a sufficiently high correlation for PCA between the CP5F subscales as follows: for G1, χ^2^ (15) = 37.373, *p* < 0.001; for G2, χ^2^ (15) = 60.117, *p* < 0.000; for G3, χ^2^ (15) = 33.439, *p* < 0.004. 

Analysis of the mean scores for CP5F (Table 1) highlighted that the lowest result was recorded by G1 with 46.768 points, and the highest result belonged to G3 with 55.600 points. Mean score differences between the three samples were as follows: −3.216 points for G1–G2, −5.616 points for G2–G3, and −8.832 points for G1–G3. As can be seen in Table 1, the total mean scores obtained for the Five-Factor Personality Questionnaire (CP5F) reflected that all three sporting age groups fell between 40 and 60 points, which represented the average range of T-score interpretation. 

Analyzing the results in Table 2, it can be seen that the arithmetic mean scores fell within the two bounds of the 95% CI. The arithmetic means of all subscales of the questionnaire were statistically significant at *p* < 0.05 for the three sporting age categories. The highest arithmetic means were recorded by G1 for the Agreeableness subscale with 48 points, by G2 for the Autonomy subscale with 54.230 points, and by G3 also for the Autonomy subscale with 57.625 points. The lowest arithmetic means were recorded by G1 for the Extraversion subscale with cu 44.692 points, by G2 for the Agreeableness subscale with 48 points, and by G3 also for the Agreeableness subscale with 54.125 points. 

**Figure 1:**
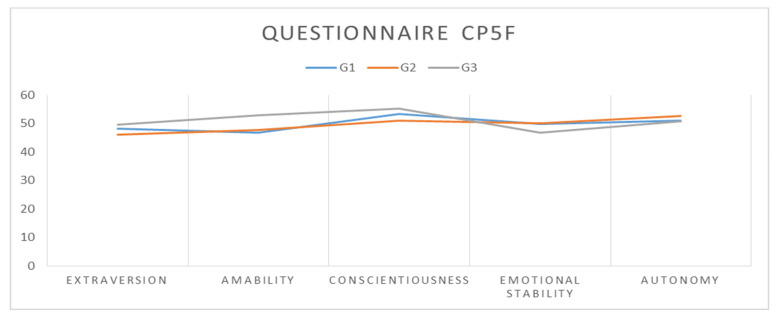
Representation of the arithmetic means recorded by the three groups for each CP5F subscale.

The arithmetic means recorded for the CP5F subscales (Table 3) showed that the ascending order of the mean scores per group was G1 with the lowest scores, followed by G2 and then G3 with the highest scores for the Extraversion, Conscientiousness, Emotional stability, and Autonomy subscales; the Agreeableness subscale was an exception because the mean scores were identical for G1 and G2, while G3 had the highest score (Figure 1). A comparative analysis of the groups taken two by two indicated an upward trend in mean scores; thus, the smallest differences were between G1 and G2, and the largest differences were between G1 and G3 for all subscales, with one exception for the Autonomy subscale. The largest difference in personality perception was recorded between G1 and G2 for the Autonomy subscale with −7.154 points, and the smallest difference for the Agreeableness subscale where the scores were identical. Analysis of the differences between G2 and G3 revealed that the largest difference was recorded for the Extraversion subscale with −7.827 points, and the smallest difference for the Autonomy subscale with −395 points. As regards the comparative analysis of groups G1–G3, the largest difference was recorded for the Extraversion subscale with −12.058 points, and the smallest difference for the Autonomy subscale with −1.549 points.

Bartlett’s Test of Sphericity values indicate a sufficiently high correlation for PCA between the Personal Autonomy Questionnaire (PAQ) subscales as follows: for G1, χ^2^ (10) = 30.988, *p* < 0.003; for G2, χ^2^ (10) = 37.364, *p* < 0.000; for G3, χ^2^ (10) = 38.607, *p* < 0.002. According to Table 4, Cronbach’s Alpha coefficient was higher than the reference threshold of 0.7, indicating high internal consistency of the Zuckerman–Kuhlman Personality Questionnaire (ZKPQ) for all three sporting age groups. Statistical values of the Kaiser–Meyer–Olkin parameter were above Kaiser’s criterion of >0.5 for all three sporting age groups. 

The total score of the Personal Autonomy Questionnaire (PAQ) reflected that all three sporting age groups fell within the average range (40–60 points) of T-score interpretation. Mean score differences between the three samples were 0.400 points for G1–G2; −1.201 points for G1–G3; and −1.601 points for G2–G3 (Table 4).

The Zuckerman–Kuhlman Personality Questionnaire (ZKPQ) is a complex tool that consists of five subscales measuring the basic factors of personality, which can be used to optimize the behavior of elite athletes. Table 5 shows that the arithmetic means of all ZKPQ subscales were statistically significant at *p* < 0.05 for the three age groups; at the same time, all these values fell within the lower and upper bounds of the 95% CI.

Analyzing the results of each sporting age category (Table 5), it was found that G1 had the highest arithmetic mean for the Activity subscale with 53.222 points and the lowest mean score for the Impulsive sensation seeking subscale with 46.666 points; G2 had the highest mean score for the Aggression–Hostility subscale with 52.538 points and the lowest mean score for the Sociability subscale with 45.923 points; G3 had the highest mean score for the Activity subscale with 55.125 points and the lowest mean score for the Neuroticism–Anxiety subscale with 46.750 points. 

A comparative analysis of the results shown in Table 6 allows us to state that the perception of the ZKPQ subscales differed according to the sporting age category; we believe that certain differences are the result of behavioral and athletic maturation. Comparing the arithmetic means of the study groups, it was found that the largest difference in the perception of the Sociability subscale was between G2 and G3 (−3.702 points), and the smallest one was between G1 and G3 (−1.403 points); for the Impulsive sensation seeking subscale, the largest difference was between G1 and G3 (−6.084 points), and the smallest one was between G1 and G2 (−0.949 points); for the Activity subscale, the largest difference was between G2 and G3 (−4.202 points), and the smallest one was between G1 and G3 (−1.903 points); for the Neuroticism–Anxiety subscale, the largest difference was between G2 and G3 (3.250 points), and the smallest one was between G1 and G2 (−0.112 points); for the Aggression–Hostility subscale, the largest difference was between G2 and G3 (1.788 points), and the smallest one was between G1 and G2 (0.250 points). 

**Figure 2:**
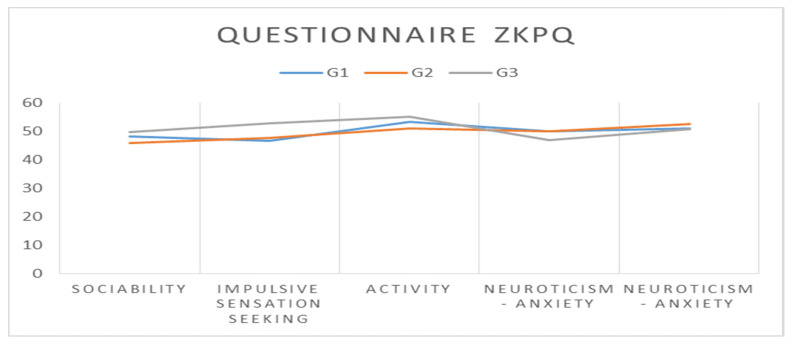
Representation of the arithmetic means recorded by the three groups for each ZKPQ subscale.

It should be noted that for the Sociability and Activity subscales, the highest mean scores were recorded by G3 and the lowest mean scores by G2; for the Impulsive sensation seeking subscale, the highest mean score was recorded by G3 and the lowest mean score by G1; for the Neuroticism–Anxiety and Aggression–Hostility subscales, the highest mean scores were achieved by G2 and the lowest mean scores by G3 (Figure 2).

Cronbach’s Alpha scores (Table 7) revealed high internal consistency of the Personal Autonomy Questionnaire (PAQ) for all three sporting age groups, the values being 0.927 for G1: 18–23 years of age; 0.897 for G2: 24–35F/40M years of age; 0.797 for G3: Over 35F/40M years of age. The same table showed that the statistical values of the Kaiser–Meyer–Olkin parameter, which was used to verify the sampling adequacy, were KMO = 0.584 for G1, KMO = 0.629 for G2, and KMO = 0.516 for G3, which were all above Kaiser’s criterion of >0.5. Bartlett’s Test of Sphericity values indicated a sufficiently high correlation for PCA between the Personal Autonomy Questionnaire (PAQ) subscales as follows: for G1, χ^2^ (10) = 6.266, *p* < 0.000; for G2, χ^2^ (10) = 75.659, *p* < 0.000; for G3, χ^2^ (10) = 66.067, *p* < 0.004.

The T-score of the Personal Autonomy Questionnaire (PAQ) showed that all three sporting age groups fell within the average range (40–60 points) of T-score interpretation. The lowest personal autonomy (PA) was recorded by G1: 18–23 years of age, with a total mean score of 48.777 points, and the highest PA was recorded by G3: Over 35F/40M years of age, with a total mean score of 56.781 points, which also reflects the level of sports experience. Mean differences between the T-scores of the three samples were as follows: 5.972 points between G2 and G1, 8.004 points between G3 and G1, and 2.032 points between G3 and G2, which indicated that an increase in sporting age category leads to an increase in the level of personal autonomy.

According to Table 8, the arithmetic means of all subscales of the questionnaire for the three groups were statistically significant at *p* < 0.05 and fell within the lower and upper bounds of the 95% CI. As regards the Personal Autonomy Questionnaire (PAQ), G1 (18–23 years of age) recorded the highest arithmetic mean for the Value autonomy subscale with 52 points and the lowest mean score for the Cognitive autonomy subscale with 45.222 points; G2 (24–35F/40M years of age) recorded the highest arithmetic mean for the Behavioral autonomy subscale with 55.769 points and the lowest mean score for the Emotional autonomy subscale with 52.692 points; G3 (Over 35F/40M years of age) achieved the highest score for the Behavioral autonomy subscale with 60.500 points, while the Emotional autonomy subscale, with 50.500 points, was less relevant for the study athletes. Overall, the subscale that recorded the highest total mean score (T-score) was the Behavioral autonomy subscale with 60.500 points for G3, and the lowest T-score was recorded by G1 for the Cognitive autonomy subscale with 45.222 points.

**Figure 3:**
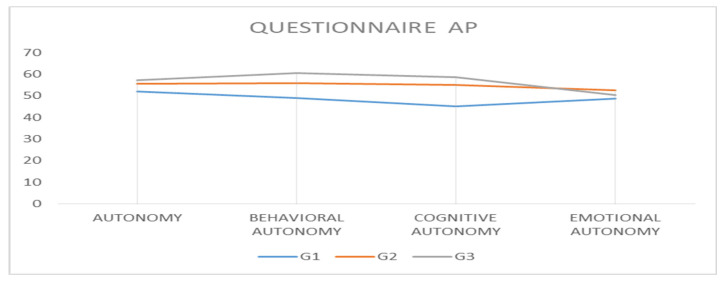
Representation of the arithmetic means recorded by the three groups for each PAQ subscale.

Mean score differences for each subscale of the Personal Autonomy Questionnaire (PAQ) revealed that the largest differences were recorded between G1 and G3 for the Value autonomy, Behavioral autonomy, and Cognitive autonomy subscales, and the smallest difference, −1.723 points, was recorded also between G1 and G3 for the Emotional autonomy subscale. The mean differences between G1 and G2 and G2 and G3 highlighted that the largest differences were recorded for the Cognitive autonomy and Behavioral autonomy subscales, and the smallest differences for the Value autonomy subscale. Analyzing the dynamics of these differences in relation to the increasing age of inclusion into the sports category, it was found (for all subscales of the questionnaire) that as the individual grew older, there was an increase in value autonomy, behavioral autonomy, cognitive autonomy, and emotional autonomy, and the dynamics of these results reflected that the largest differences were between G1 and G3, but the differences between G1 and G2 were also larger than those between G2 and G3 for all subscales (Table 9, Figure 3).

The ANOVA data (Table 10) facilitated the identification of statistically significant differences between the mean scores of the three groups structured into sporting age categories for all three questionnaires selected to be used in this study. The results confirmed significant differences between the total scores of the three study groups for all three questionnaires.

## 4. Discussion

The present study aimed at identifying the differences in personality and autonomy factors in order to identify the differences in perception for three sporting age categories of bodybuilding athletes. We believe that the results obtained and analyzed in this study will contribute to a better understanding and the expansion of knowledge about the possibilities of developing personality traits in relation to the sport practiced and the bodybuilding-specific differences by sporting age category. The main results of the study fall within the research directions specific to sports psychology, pointing out particular psychological aspects typical to competitive bodybuilders. Psychological profiles can be developed by highlighting the dynamics of perceived personality factors according to the three sporting age categories, which are regulated for international competitions as follows: junior: 18–23 years old; senior: 25–35 years old for female and 25–40 years old for male; masters: over 35 years old for female and 40 for male. The results of our study emphasize numerous differences between the three age categories of competitive bodybuilders, and we believe that these differences arise from athletic experience, competitive experience, and psychological and behavioral maturation in relation to the specifics of the sport practiced.

The concerns of experts in sports psychology and specialists in competitive sports have focused on how personality factors determine and influence the optimization of sports performance [75,76,77,78]. According to our study, personality factors are influenced by athletic experience, individual characteristics, and the specifics of the sport practiced. The results of the present study complement the information of previous studies assessing personality factors based on both the specifics of the sport practiced and individual and age characteristics [66,79,80]. In line with our study, a previous study carried out on 881 subjects that investigated the Big Five personality traits between athletes and non-athletes concluded that successful athletes had higher scores for each subscale and total scores than those who did not perform [81]. Another study carried out on 87 adult competitive athletes, male and female, analyzed their resilience and optimism, as well as the relationship between these personality parameters and the sports category, the gender, and the duration of the sports experience; the conclusions highlighted that there were no gender differences, and the level of experience and the sports category influence the link between resilience and optimism, the results being higher for the most experienced and senior [82]. Another study carried out in 2015 on male athletes who practice bodybuilding highlighted that by combining psychological and biological factors, the level of self-esteem and the appearance of male dysmorphia (MD) can be influenced [83]. A complex study aimed at analyzing eating behavior, anthropometric parameters, and general psychological factors included 74 competitive and non-competitive male bodybuilders, and the results did not highlight significant differences regarding psychological factors, but they did highlight differences regarding eating behaviors [84]. The results recorded in this study confirm the findings of previous studies, which demonstrated that achieving success in sport and implicitly optimizing the potential for sports performance depend on the athlete’s personality and experience, both of them reflected in high levels of sociability, activity, autonomy, and emotional stability [33,80]. An important concern of sports field specialists, including coaches and sports psychologists, aimed at identifying the relationship between personality factors in the sense of their modeling for achieving sports success and the determining factors for obtaining champion status. Thus, based on the criteria of the Big Five Model, a study that included 60 sportswomen from 12 disciplines was completed with personality profiles of the champions in combat sports, identifying 45 champions at the international competitive level at the European level and world, and registering statistically significant differences between the disciplines of combat athletes in the dimensions of neuroticism, extraversion, agreeableness, and conscientiousness [85]. The results of the previous study were completed and confirmed by another study conducted on 1260 others through which major differences were identified between champion athletes and regular athletes [86]. In the same direction of research, another study that analyzed 806 competitive athletes aimed at the interrelation between dark personality (narcissism and Machiavellianism) and competitiveness; the authors concluded that dark personality traits are influenced by individual peculiarities, self-perception of psychological peculiarities, and the competitive sports environment [87]. In 2018, a study was carried out on 133 male and 119 female bodybuilding practitioners, focused on identifying gender differences regarding the motivations in the weight training process. The study identified significant differences; thus, the male sample was motivated by the increase in muscle mass, while the female sample was motivated by health, well-being, leisure, and socialization [88].

Extraversion was another personality factor studied by sports psychologists, who emphasized that bodybuilding and fitness athletes with high levels of extraversion were more susceptible to exercise addiction [89,90]. The results of our study also enhanced the level of knowledge achieved in other studies aimed at identifying the aggression–hostility levels depending on the specifics of the sport; thus, given that bodybuilding is a non-aggressive sport, the level of this parameter was relatively low for the investigated athletes compared to athletes involved in contact sports [91,92,93,94]. Moreover, aggression usually decreases with increasing experience and sporting age.

Several studies have examined the relationship between personality and competitive anxiety, concluding that the type of sport and the gender of athletes are determining factors for the level of sports anxiety [95,96,97]. Our study also demonstrated that anxiety levels decrease in competitive bodybuilding athletes as their competitive experience and sporting age increase. A study done on competitive bodybuilders highlighted that anxiety traits increase in relation to sports categories and implicitly with the requirements of sports training and participation in competitions [98]. A study carried out in 2022 aimed to compare the five personality factors and anxiety between individual athletes (114 subjects) and athletes from team sports (123 subjects); the results showed that there were no significant differences between the two samples regarding personality factors, but anxiety was much higher in individual athletes [94]. To enhance the analysis framework of athletes’ personalities, another dimension of personality addressed in our study was personal autonomy as a predictor of self-determination and self-control of both sports activity and sporting life. The present study showed that as athletic experience and sporting age increase, the components of autonomy improve, which we consider to have an important role in the optimization of sports performance in bodybuilding. Numerous studies on autonomy have been carried out to identify autonomy factors depending on the type of sport practiced [99,100,101], the gender of athletes [102], the manifestation of self-determination and motivation in recreational and competitive athletes [103,104], and the level of sports skills in champions [52,103]. Research on personal autonomy in athletes has also focused on identifying intrinsic and extrinsic motivations for practicing a certain type of sport and improving sports performance [105,106,107,108]. The results of our study highlight that cognitive autonomy, behavioral autonomy, and value autonomy increase directly proportionally to athletic experience and sporting age, while emotional autonomy is lower in the junior group (G1) and masters group (G3) compared to the senior group (G2). We believe that the positive dynamics of the results is directly influenced by the athletes’ levels of self-determination and intrinsic and extrinsic motivation for improving sports performance in bodybuilding. Emotional autonomy reflects (independently of the formation and manifestation of feelings and the presence of fluctuations generated by personal and athletic motivations) the sporting lifestyle and contexts [108]. In line with our study, we mention that the study carried out in 2020 on 114 bodybuilders highlighted that extraversion and narcissism can have a major influence on the formation of behaviors dependent on the practice of physical exercises [80]. Our study aligns with another study conducted on 600 athletes, including 56 champions and 544 athletes from individual disciplines, to which the Big Five Model for identifying relevant personality factors (NEO-FFI) was applied, concluding that sports champions have a lower level of neuroticism, but the level of extraversion, agreeableness, and conscientiousness is significantly higher than in other athletes [51]. Another similar study carried out on 218 bodybuilders focused on identifying personality differences between the groups of competitive and non-competitive athletes highlighted significant differences regarding reasoning, abstraction, and perfectionism [109]. Studies on bodybuilders have highlighted significant differences regarding personality traits and autonomy between competitive and non-competitive bodybuilders [110,111,112,113]. A comparative study between competitive and non-competitive bodybuilders carried out on 78 athletes came to the conclusion that no significant differences could be identified between samples regarding psychosocial behavioral characteristics [26]. Competitive athletics can be practiced by athletes who adopt a disciplined lifestyle due to the regularity and duration of training in order to adapt their motor and psychological potential to the growing demands of bodybuilding. Understanding the mechanisms and contexts of development of personality factors in competitive athletes requires complex approaches towards identifying how they interact and correlate in order to provide the mental and behavioral basis necessary for the practice of bodybuilding. 

The strengths of the study are as follows: identification of the differences between the three sporting age samples of competitive bodybuilders included in the study; the inclusion in the study of all successful competitive athletes at the 2021 Romanian national championships; and the complex analysis of ten personality factors and four personal autonomy factors. Limitations of the study include the relatively low number of elite bodybuilding athletes in our country; unequal gender distribution of athletes into the three study groups; and the inclusion in the current study only of competitive bodybuilders who obtained the title of national champion and participated with good results in international competitions at the Balkan, European, and world levels.

## 5. Conclusions

The results of the study confirm the main hypothesis, which is that there are significant differences in the perception of the factors of personality and autonomy of competitive bodybuilders in relation to the sports age category and sports experience. According to these results, the following personality factors recorded higher and higher scores in direct relation with the increase in sporting age: extraversion, agreeableness, conscientiousness, emotional stability, autonomy, and impulsive sensation seeking. Personality factors such as sociability and activity recorded higher values in the junior and masters groups compared to the senior group. The aggression–hostility and neuroticism–anxiety personality factors had also lower scores in the junior and masters groups compared to the senior group. Analyzing the results of the Personal Autonomy Questionnaire (PAQ), it was seen that the value autonomy, behavioral autonomy, and cognitive autonomy factors increased with the increase in sporting age category, while the emotional autonomy factor recorded the lowest scores in the junior and masters groups and the highest score in the senior group. Achieving performance in bodybuilding depends on the level of development and manifestation of personality and autonomy factors, and their dynamics are influenced by age characteristics, athletic experience, and the type of sport practiced. The scientific management of sports training requires from the staff the knowledge of athletes in their complexity, and the factors of personality and autonomy are important in understanding the personality and behaviors of athletes. The identification of the differences in the perception of competitive bodybuilders depending on the age category and sports experience contributes to the identification by sports psychologists and coaches of the most effective strategies for approaching the psychological counseling process. The identification of the common aspects and the differences of perception of the factors of personality and autonomy are current concerns of the specialists and researchers in the field of sports psychology, and the expansion of the knowledge of these aspects depending on the training and competition characteristics of the sport, the sports experience, the category of sports age, such as the breakdown of specialists from the technical staff, etc., can contribute to the optimization of the sports training process.

## Figures and Tables

**Table 1:** Statistical analysis of the data for the Five-Factor Personality Questionnaire (CP5F).

Age Group Category	Cronbach’s Alpha	KMO	Bartlett’s Test of Sphericity			Total Mean Score (T-Score)
			χ^2^	df	*p*	
G1: 18–23	0.777	0.519	37.373	15	0.001	46.768
G2: 24–35F/40M	0.753	0.680	60.117	15	0.000	49.984
G3: Over 35F/40M	0.794	0.516	33.439	15	0.004	55.600

KMO—Kaiser–Meyer–Olkin Measure of Sampling Adequacy, χ^2^—Chi-square, df—degree of freedom, *p*—level of probability, F—female, M—male.

**Table 2:** Descriptive statistics of the results for the Five-Factor Personality Questionnaire (CP5F) subscales.

Age Group Category	Subscale	Min	Max	Mean	SD	*t*	*p*	95% CI	
								Lower	Upper
G1: 18–23	Extraversion	31.00	58.00	44.692	8.67	18.578	0.000	39.45	49.93
	Agreeableness	26.00	59.00	48.000	8.50	20.349	0.000	42.86	53.13
	Conscientiousness	30.00	68.00	46.923	10.73	15.760	0.000	40.43	53.41
	Emotional stability	31.00	69.00	47.153	12.89	13.184	0.000	39.36	54.94
	Autonomy	20.00	69.00	47.076	13.66	12.421	0.000	38.81	55.33
G2: 24–35F/40M	Extraversion	31.00	71.00	48.923	12.63	13.964	0.000	41.28	56.55
	Agreeableness	15.00	77.00	48.000	14.05	12.310	0.000	39.50	56.49
	Conscientiousness	25.00	73.00	49.846	11.21	16.023	0.000	43.06	56.62
	Emotional stability	35.00	72.00	48.923	11.72	15.048	0.000	41.83	56.00
	Autonomy	37.00	72.00	54.230	11.84	16.504	0.000	47.07	61.39
G3: Over 35F/40M	Extraversion	37.00	69.00	56.750	11.08	14.486	0.000	47.48	66.01
	Agreeableness	40.00	63.00	54.125	7.93	19.290	0.000	47.49	60.75
	Conscientiousness	37.00	65.00	55.250	8.77	17.800	0.000	47.91	62.58
	Emotional stability	40.00	74.00	54.250	10.85	14.130	0.000	45.17	63.32
	Autonomy	43.00	75.00	57.625	10.76	15.134	0.000	48.62	66.62

G—group, F—female, M—male, Min—minimum, Max—maximum, SD—standard deviation, *t*—Student test value, *p*—level of probability, CI—confidence interval.

**Table 3:** Mean differences between groups for the Five-Factor Personality Questionnaire (CP5F) subscales.

Subscale	Age Group Category	Mean			
		Mean	Mean Dif. G1–G2	Mean Dif. G2–G3	Mean Dif. G1–G3
Extraversion	G1: 18–23	44.692	−4.231	−7.827	−12.058
	G2: 24–35F/40M	48.923			
	G3: Over 35F/40M	56.750			
Agreeableness	G1: 18–23	48.000	-	−6.125	−6.125
	G2: 24–35F/40M	48.000			
	G3: Over 35F/40M	54.125			
Conscientiousness	G1: 18–23	46.923	−2.923	−5.404	−8.327
	G2: 24–35F/40M	49.846			
	G3: Over 35F/40M	55.250			
Emotional stability	G1: 18–23	47.153	−1.770	−5.327	−7.097
	G2: 24–35F/40M	48.923			
	G3: Over 35F/40M	54.250			
Autonomy	G1: 18–23	47.076	−7.154	−3.395	−1.549
	G2: 24–35F/40M	54.230			
	G3: Over 35F/40M	57.625			

Mean dif.—mean difference, G—group, F—female, M—male.

**Table 4:** Statistical analysis of the data for the Zuckerman–Kuhlman Personality Questionnaire (ZKPQ).

Age Group Category	Cronbach’s Alpha	KMO	Bartlett’s Test of Sphericity			Total Mean Score (T-Score)
			χ^2^	df	*p*	
G1: 18–23	0.758	0.691	30.988	10	0.003	49.799
G2: 24–35F/40M	0.772	0.544	37.364	10	0.000	49.399
G3: Over 35F/40M	0.793	0.647	38.607	10	0.002	51.000

KMO—Kaiser–Meyer–Olkin Measure of Sampling Adequacy, χ^2^ = Chi-square, df—degrees of freedom, *p*—level of probability, F—female, M—male.

**Table 5:** Descriptive statistics of the results for the Zuckerman–Kuhlman Personality Questionnaire (ZKPQ) subscales.

Age Group Category	Subscale	Min	Max	Mean	SD	*t*	*p*	95% CI	
								Lower	Upper
G1: 18–23	Sociability	36.00	69.00	48.222	12.31	11.746	0.000	38.75	57.68
	Impulsive sensation seeking	33.00	56.00	46.666	7.58	18.463	0.000	40.83	52.49
	Activity	28.00	67.00	53.222	11.36	14.047	0.000	44.48	61.95
	Neuroticism–Anxiety	37.00	63.00	49.888	9.94	15.053	0.000	42.24	57.53
	Aggression–Hostility	31.00	64.00	51.000	10.93	13.996	0.000	42.59	59.40
G2: 24–35F/40M	Sociability	32.00	56.00	45.923	7.83	21.129	0.000	41.18	50.65
	Impulsive sensation seeking	30.00	68.00	47.615	10.75	15.968	0.000	41.11	54.11
	Activity	32.00	67.00	50.923	10.42	17.620	0.000	44.62	57.21
	Neuroticism–Anxiety	33.00	68.00	50.000	11.86	15.191	0.000	42.82	57.17
	Aggression–Hostility	32.00	79.00	52.538	12.69	14.917	0.000	44.86	60.21
G3: Over 35F/40M	Sociability	34.00	64.00	49.625	9.92	14.139	0.000	41.32	57.92
	Impulsive sensation seeking	33.00	68.00	52.750	12.32	12.105	0.000	42.44	63.05
	Activity	43.00	65.00	55.125	7.69	20.253	0.000	48.68	61.56
	Neuroticism–Anxiety	31.00	65.00	46.750	11.88	11.122	0.000	36.81	56.68
	Aggression–Hostility	37.00	67.00	50.750	10.30	13.928	0.000	42.13	59.36

G—group, F—female, M—male, Min—minimum, Max—maximum, SD—standard deviation, *t*—Student test value, *p*—level of probability, CI—confidence interval.

**Table 6:** Mean differences between groups for the Zuckerman–Kuhlman Personality Questionnaire (ZKPQ) subscales.

Subscale	Age Group Category	Mean			
		Mean	Mean Dif. G1–G2	Mean Dif. G2–G3	Mean Dif. G1–G3
Sociability	G1: 18–23	48.222	2.299	−3.702	−1.403
	G2: 24–35F/40M	45.923			
	G3: Over 35F/40M	49.625			
Impulsive sensation seeking	G1: 18–23	46.666	−0.949	−5.135	−6.084
	G2: 24–35F/40M	47.615			
	G3: Over 35F/40M	52.750			
Activity	G1: 18–23	53.222	2.299	−4.202	−1.903
	G2: 24–35F/40M	50.923			
	G3: Over 35F/40M	55.125			
Neuroticism–Anxiety	G1: 18–23	49.888	−0.112	3.250	3.138
	G2: 24–35F/40M	50.000			
	G3: Over 35F/40M	46.750			
Aggression–Hostility	G1: 18–23	51.000	−1.538	1.788	0.250
	G2: 24–35F/40M	52.538			
	G3: Over 35F/40M	50.750			

Mean dif.—mean difference, G—group, F—female, M—male.

**Table 7:** Statistical analysis of the data of the Personal Autonomy Questionnaire (PAQ).

Age Group Category	Cronbach’s Alpha	KMO	Bartlett’s Test of Sphericity			Total Mean Score (T-Score)
			χ^2^	df	*p*	
G1: 18–23	0.927	0.584	69.266	10	0.000	48.777
G2: 24–35F/40M	0.897	0.629	75.659	10	0.000	54.749
G3: Over 35F/40M	0.797	0.516	66.067	10	0.004	56.781

KMO—Kaiser–Meyer–Olkin Measure of Sampling Adequacy, χ^2^—Chi-square, df—degrees of freedom, *p*—level of probability, G—group, F—female, M—male.

**Table 8:** Descriptive statistics of the results of the Personal Autonomy Questionnaire (PAQ) subscales.

Age Group Category	Subscale	Min	Max	Mean	SD	*t*	*p*	95% CI	
								Lower	Upper
G1: 18–23	Value autonomy	39.00	68.00	52.000	10.85	14.376	0.000	43.65	60.34
	Behavioral autonomy	28.00	68.00	49.111	14.35	10.262	0.000	38.07	60.14
	Cognitive autonomy	26.00	65.00	45.222	13.96	9.717	0.000	34.48	55.95
	Emotional autonomy	31.00	61.00	48.777	11.08	13.197	0.000	40.25	57.30
G2: 24–35F/40M	Value autonomy	38.00	68.00	55.538	8.43	23.748	0.000	50.44	60.63
	Behavioral autonomy	37.00	73.00	55.769	10.25	19.605	0.000	49.57	61.96
	Cognitive autonomy	39.00	70.00	55.000	8.92	22.218	0.000	49.60	60.39
	Emotional autonomy	27.00	66.00	52.692	11.36	16.723	0.000	45.82	59.55
G3: Over 35F/40M	Value autonomy	43.00	77.00	57.375	13.38	12.125	0.000	46.18	68.56
	Behavioral autonomy	54.00	77.00	60.500	7.48	22.867	0.000	54.24	66.75
	Cognitive autonomy	48.00	70.00	58.750	7.61	21.833	0.000	52.38	65.11
	Emotional autonomy	31.00	64.00	50.500	10.79	13.229	0.000	41.47	59.52

G—group, F—female, M—male, Min—minimum, Max—maximum, SD—standard deviation, *t*—Student test value, *p*—level of probability, CI—confidence interval.

**Table 9:** Mean differences between groups for the Personal Autonomy Questionnaire (PAQ) subscales.

Subscale	Age Group Category	Mean			
		Mean	Mean Dif. G1–G2	Mean Dif. G2–G3	Mean Dif. G1–G3
Value autonomy	G1: 18–23	52.000	−3.538	−1.837	−5.375
	G2: 24–35F/40M	55.538			
	G3: Over 35F/40M	57.375			
Behavioral autonomy	G1: 18–23	49.111	−6.658	−4.731	−11.389
	G2: 24–35F/40M	55.769			
	G3: Over 35F/40M	60.500			
Cognitive autonomy	G1: 18–23	45.222	−9.778	−3.750	−13.528
	G2: 24–35F/40M	55.000			
	G3: Over 35F/40M	58.750			
Emotional autonomy	G1: 18–23	48.777	−3.915	−2.192	−1.723
	G2: 24–35F/40M	52.692			
	G3: Over 35F/40M	50.500			

Mean dif.—mean difference, G—group, F—female, M—male.

**Table 10:** ANOVA—analysis of variance between the three study groups.

Questionnaire	Total Score	df	F	*p*
PA	54.435	2	4.623	0.005
CP5F	50.786	2	3.172	0.002
ZKPQ (5 subscales)	50.066	2	4.264	0.003

df—degrees of freedom, F—F-test value, *p*—level of probability.

## Data Availability

Not applicable.
